# Metadichol®: A Novel Nanolipid Formulation That Inhibits SARS-CoV-2 and a Multitude of Pathological Viruses In Vitro

**DOI:** 10.1155/2022/1558860

**Published:** 2022-01-15

**Authors:** Palayakotai R. Raghavan

**Affiliations:** Nanorx Inc., P.O. Box 131 Chappaqua, NY 10514, USA

## Abstract

Increasing outbreaks of new pathogenic viruses have promoted the exploration of novel alternatives to time-consuming vaccines. Thus, it is necessary to develop a universal approach to halt the spread of new and unknown viruses as they are discovered. One such promising approach is to target lipid membranes, which are common to all viruses and bacteria. The ongoing severe acute respiratory syndrome coronavirus 2 (SARS-CoV-2) pandemic has reaffirmed the importance of interactions between the virus envelope and the host cell plasma membrane as a critical mechanism of infection. Metadichol®, a nanolipid emulsion of long-chain alcohols, has been demonstrated as a strong candidate that inhibits the proliferation of SARS-CoV-2. Naturally derived substances, such as long-chain saturated lipid alcohols, reduce viral infectivity, including that of coronaviruses (such as SARS-CoV-2) by modifying their lipid-dependent attachment mechanism to human host cells. The receptor ACE2 mediates the entry of SARS-CoV-2 into the host cells, whereas the serine protease TMPRSS2 primes the viral S protein. In this study, Metadichol® was found to be 270 times more potent an inhibitor of TMPRSS2 (EC_50_ = 96 ng/mL) than camostat mesylate (EC_50_ = 26000 ng/mL). Additionally, it inhibits ACE with an EC_50_ of 71 ng/mL, but it is a very weak inhibitor of ACE2 at an EC_50_ of 31 *μ*g/mL. Furthermore, the live viral assay performed in Caco-2 cells revealed that Metadichol® inhibits SARS-CoV-2 replication at an EC_90_ of 0.16 *μ*g/mL. Moreover, Metadichol® had an EC_90_ of 0.00037 *μ*M, making it 2081 and 3371 times more potent than remdesivir (EC_50_ = 0.77 *μ*M) and chloroquine (EC_50_ = 1.14 *μ*M), respectively.

## 1. Introduction

Currently, there is an increasing need to develop broad-spectrum antimicrobial agents that can inactivate human pathogens, such as bacteria and viruses. Moreover, rapid development of antimicrobial resistance in microorganisms has propelled the development of targeted drugs. The most recent trigger is the fear of a future pandemic caused by poorly studied novel virulent strains, such as the severe acute respiratory syndrome coronavirus 2 (SARS-CoV-2).

### 1.1. Background Information on SARS-CoV-2

The ongoing COVID-19 pandemic caused by SARS-CoV-2 [1] has created global havoc within a few months of its emergence. Medically controlling the rapid viral spread by utilizing specific antivirals and vaccines is expensive and time-consuming and compromises on the safety and efficacy of the treatment. Thus, an alternative approach is to test compounds that have already been proven to be effective and safe against SARS-CoV-2. In this study, camostat mesylate (a 35-year-old Japanese drug), Avigan (another Japanese drug), and remdesivir (Gilead Science Inc.) were evaluated for this purpose [2].

The SARS-CoV-2 virus enters a host cell by interacting with the transmembrane protease serine 2 (TMPRSS2) [3], a serine protease, and the angiotensin-converting enzyme 2 (ACE2) present on the host cell [4]. Thus, inhibiting TMPRSS2 is the key to blocking the virus from binding to ACE2, hindering the mechanism underlying its entry into the host cell.

The serine protease TMPRSS2 is responsible for priming the spike proteins of SARS-CoV and Middle East respiratory syndrome-related coronavirus. Studies have demonstrated that the TMPRSS2 inhibitor camostat mesylate (CM) inhibits SARS-CoV-2 in a mouse model [5, 6]. Furthermore, Hoffmann et al. [7] determined that SARS-CoV-2 requires TMPRSS2 for entry into host cells, as they demonstrated that CM blocked viral entry into the lungs. However, to date, there are no clinical data on the use of CM to treat COVID-19 patients.

The other host receptor essential viral entry into the host cells is the transmembrane protein ACE2, as the spike (S) protein on the exterior of the SARS-CoV-2 viral envelope binds to ACE2. However, as ACE2 also regulates blood pressure and blood volume, blocking ACE2 is detrimental to the health of the host. Thus, an ideal approach to inhibit viral entry is to inhibit TMPRSS2.

### 1.2. Lipids and Viruses

The viral lipid envelope is crucial for both viral stability and infection. For example, substances, such as phospholipases, organic solvents, and surfactants (e.g., soaps), that affect this envelope have been demonstrated to affect viral infectivity as well [8]. Thus, disintegrating this envelope can prevent transmission of the virus to a new host. Furthermore, active ingredients in cleaning agents, wipes, and tissues target the viral lipid envelope to render the virions nonviable. Snipes et al. [9] reported that saturated alcohols with chain lengths ranging from 10 to 14 carbons can inactivate viruses. They also established that inactivation of these enveloped viruses using lipids varied greatly depending on both the nature of the lipid and the type of the virus. Hilmarsson et al. [10–12] studied the virucidal effects of medium- and long-chain fatty alcohols (8–18 carbons) and those of corresponding lipids against the herpes simplex viruses (HSV-1 and HSV-2), respiratory syncytial virus, human parainfluenza virus type 2, and enveloped viruses at various concentrations, time points, and pH levels. They found that after a 10-minute incubation at 37°C, 14 of the tested lipids caused a significant reduction (100000-fold or more) in HSV titer at 10 mM concentrations. Additionally, a pH of 4.2 caused a more rapid inactivation of HSV-1 in one minute than higher pH values (pH 7) did. Thus, it can be deduced that these long-chain alcohols possibly penetrate the viral envelope hydrophobically, making the envelope permeable to small molecules and thus inactivating the virus. However, the degree of penetration into the lipid membranes is based on the chain length of the alcohols compared with the thickness of the membrane [13].

Metadichol® is a lipid formulation of long-chain alcohols, containing C26, C28 (more than 80%), and C30 [14]. Previous studies have demonstrated that Metadichol® inhibits viruses both *in vitro* and *in vivo* [15–17]. Thus, in this study, we evaluated the inhibitory effect of Metadichol, a nanoemulsion, against ACE2, angiotensin-converting enzyme (ACE), and TMPRSS2 and tested its efficacy in a SARS-CoV-2 antiviral assay.

## 2. Materials and Methods

All assays were conducted on a fee-for-service contract basis and outsourced to bioanalytical testing companies worldwide. The SARS-CoV-2 antiviral assays were performed in a biosafety level 3 facility at the Anti-Viral Research Institute Utah State University, Logan, Utah, USA. Other assays were performed in the Infectious Disease Research Facility at the Southern Research Institute in Frederick, Maryland, USA, and IBT Bio-Services in Rockville, Maryland, USA. Additionally, the ACE2, ACE, and TMPRSS2 assays were carried out by Skanda Life Sciences Pvt. Ltd. in Bangalore, India.

### 2.1. Antiviral Assay

Metadichol was serially diluted into eight half-log dilutions in a test medium (minimum essential media supplemented with 2% fetal bovine serum and 50 *μ*g/mL of gentamicin) to obtain a high starting test concentration of 100 *μ*g/mL. Each dilution was added to five wells in a 96-well plate containing Caco-2 cells (80–100% confluency). Subsequently, three wells of each dilution were inoculated with SARS-CoV-2, whereas the other two wells were uninoculated (as cytotoxicity controls). Additionally, six wells were inoculated with the virus but left untreated (viral controls), whereas six wells were uninoculated and untreated (cell controls). For the next five days, the lowest possible multiplicity of infection value for SARS-CoV-2 that led to >80% cytopathic effect (CPE) in the host cells was evaluated. The SARS-CoV-2-specific protease inhibitor M128533 was also tested in parallel as a positive control. The plates were incubated at 37 ± 2°C in 5% CO_2_. Once the viral control cells exhibited maximum CPE on the third day postinfection, neutral red dye was added in the wells for approximately 120 ± 15 min. The dye in the supernatant was removed, and the wells were rinsed with PBS. Subsequently, the incorporated dye was extracted using a 50 : 50 ratio of Sorensen citrate buffer to ethanol for >30 min. The optical densities were measured at 540 nm using a spectrophotometer. These values (in percentage) were normalized with those of the cell controls, and the cytotoxic concentration of a compound that caused 50% cell death (CC_50_) in the absence of the virus was calculated by using regression analysis. The selective index (SI) was obtained by dividing the CC_50_ value with the EC_90_ value. These results are listed in Table 1.

To perform the viral yield reduction assay, the supernatant was collected from wells corresponding to each concentration of Metadichol on day three postinfection. Following this, neutral red was added to the wells (3 wells of each concentration pooled) and the viral titer was measured. This was done by conducting a standard endpoint dilution CCID_50_ assay in Vero 76 cells and by calculating the viral titer using the Reed-Muench (1938) equation [18]. The concentration of a compound that reduced the viral yield by one log_10_ was calculated by using regression analysis (EC_90_).

As shown in Table 2, the viral reduction assay did not follow a typical dose-response curve, as viral reduction was observed at 0.3 *μ*g/mL and 3.2 *μ*g/mL, but no reduction was observed at 1 *μ*g/mL. Thus, it was assumed that the viral breakthrough at 1 *μ*g/mL was an outlier. The calculated SI was 20 (Table 1), indicating an EC_90_ of 0.15 *μ*g/mL.

Similarly, the experimental results for other viruses carried out by various laboratories in Vero cells are depicted in Tables 3 and 4.  Table 5 is the list of all viruses inhibited by Metadichol®.

### 2.2. TMPRSS2 Inhibition Assay

The TMPRSS2 protein was purified from LNCaP cells (obtained from American Tissue Culture Collection) and used as an enzyme source. A reaction mixture containing the purified TMPRSS2 protease in Tris-buffered saline was prepared with or without the test samples or protease inhibitor (concentrations ranged from 1.56 to 100 ng/mL for both). The reaction mixture was incubated for 10 min at 37°C. Subsequently, 1 *μ*L of 10 mM of the fluorogenic trypsin substrate Cbz-Gly-Gly-Arg-AMC was added to the reaction mixture and incubated for 2 min at 37°C. The kinetic fluorescence values were measured at an excitation wavelength of 383 nm and an emission wavelength of 455 nm at 10 minutes using SpectraMax i3x (Molecular Devices, San Jose, CA USA). The inhibitory effects of the test samples were determined by calculating the changes in their RFU (Relative Fluorescence Units). Moreover, CM (camostat mesylate) (sourced from Cayman Chemicals) was used at concentrations ranging from 1.56 to 100 nm (nanomolar) and was used as a positive control for TMPRSS2 inhibition.

### 2.3. ACE2 Inhibition Assay

The ACE2 Inhibitor Screening Assay Kit (catalog no. 79923, BPS Biosciences, San Diego, CA, USA) was used to measure the exopeptidase activity of ACE2 and to evaluate the inhibitory effect of Metadichol and DX600 (control) on ACE2. The inhibitory activities of these compounds were measured by the intensity of the fluorescence emitted upon cleavage of the chromogenic substrate.

Enzyme (ACE2) stocks were prepared using the supplied kit. Subsequently, 20 *μ*L of the enzyme solution (0.5 ng/*μ*L) was added to all the wells designated for the assay. The potent ACE2 inhibitor DX600 was used as a positive control for ACE2 inhibition at concentrations ranging from 0.0156 to 1 *μ*g/mL. Additionally, the inhibitory effects of the test samples were tested at concentrations ranging from 0.125 to 40 *μ*g/mL. Thereafter, 5 *μ*L of the inhibitors was added to the wells containing the enzyme solution. The resultant reaction mixture was incubated at room temperature for 5 min. Postincubation, 25 *μ*L of the ACE2 substrate was added to the wells and incubated for 1 h at room temperature. The RFU upon the cleavage of the substrate was measured at an excitation wavelength of 555 nm and an emission wavelength of 585 nm using the SpectraMax i3x (Molecular Devices). The IC_50_ values were calculated based on these measurements.

### 2.4. ACE Inhibition Assay

The inhibitory activity of the test samples against ACE was assessed using the angiotensin I-converting enzyme (ACE) Fluorometric Activity Assay Kit (cat. no. CS0002) as per the manufacturer's instructions, with slight modifications.

#### 2.4.1. Sample Preparation

1. The ACE-positive control was used as the main enzyme source for the assay, and a working stock solution was prepared by diluting ACE 250-fold in the assay buffer
2. Sample stock solutions of 5 mg/mL were used to obtain various desired concentrations of the test samples
3. The final volumes of all the test samples were 25 *μ*L (2× concentration)

#### 2.4.2. Assay Procedure

All reagents were equilibrated at 37°C for 5 min before performing the assay. Freshly prepared ACE enzyme solution (25 *μ*L) was added to test sample solutions (25 *μ*L of different 2× concentrations) in 96-well flat-bottom black plates. This reaction mixture was gently mixed using a pipette and incubated for 5 min at room temperature. The reaction was initiated by adding 50 *μ*L of 100-fold diluted substrate to a final reaction volume of 100 *μ*L and incubated for 5 min at room temperature. Subsequently, the fluorescence intensity of each reaction was measured at an excitation wavelength of 320 nm and an emission wavelength of 405 nm.

Captopril was used as a positive control at various concentrations.

## 3. Results and Discussion

As summarized in Tables 1 and 2, Metadichol had a direct antiviral effect against SARS-CoV-2 in Caco-2 cells at an EC_90_ of 0.15 *μ*g/mL (0.00026 *μ*M). Thus, this demonstrated that Metadichol was 2000-fold more effective as an antiviral agent than remdesivir (EC_50_ 0.77 *μ*M) and 3000-fold more potent than hydroxychloroquine phosphate (EC_50_ 1.13 *μ*M) [19].

A previously published work [15] of antiviral data against other viruses is presented in Tables 3 and 4. The raw data depict the cytotoxicity of Metadichol in the absence of a virus in Vero cells measured using a neutral red assay. No viral CPE value was reported when the “cytotoxicity” was >75%. These results may suggest that Metadichol is cytotoxic to cells at concentrations above 5 *μ*g/mL. However, 0Metadichol is not toxic, as the LD_50_ value is 5000 mg/kg in rats [20–22]. It is likely that Metadichol mimics characteristics of soaps and disrupts the lipid membrane of virus at higher concentrations, whereas it neutralizes the virus by a different mechanism at lower concentrations. Additionally, Metadichol selectively targets cancer cells in Caco-2 cells [23] and cancer cell lines MIA-PaCa, COLO 205, and Panc1 where Metadichol was cytotoxic to all these cell lines above 1 *μ*g/mL. It is selectively cytotoxic at 10 *μ*g/mL in leukemia cells [24].

**Figure 1:**
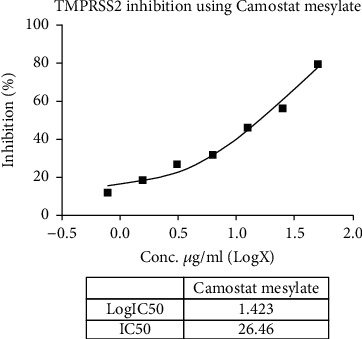
Inhibition of TMPRSS2 by camostat mesylate (control).

**Figure 2:**
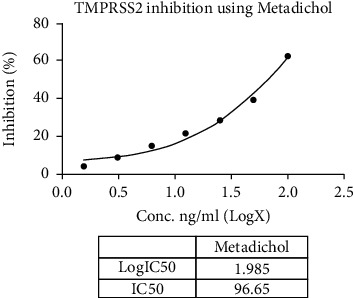
Inhibition of TMPRSS2 by Metadichol.

**Figure 3:**
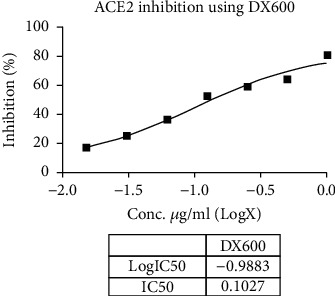
Inhibition of ACE2 by DX600 (control).

**Figure 4:**
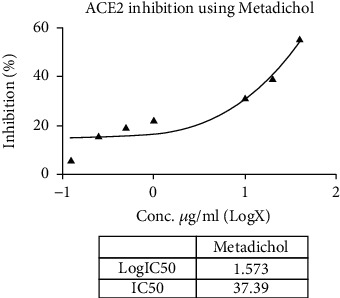
Inhibition of ACE2 by Metadichol.

**Figure 5:**
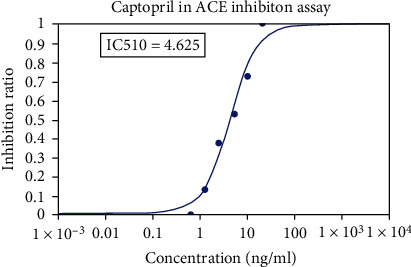
Inhibition of ACE by captopril (control).

**Figure 6:**
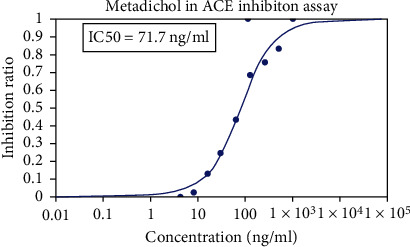
Inhibition of ACE by Metadichol.

Metadichol also inhibited TMPRSS2 (Table 6 and Figures 1 and 2) and was 270-fold more potent than CM [25]. Nevertheless, for all practical purposes, it does not inhibit ACE2 (Table 7 and Figures 3 and 4) and inhibits TMPRSS2 which is needed for the virus to bind to ACE 2. Thus, the reported results provide a gateway to effective and safe therapies for COVID-19 patients. On the other hand, Metadichol did inhibit ACE (Table 8 and Figures 5 and 6). Inhibition of ACE, a blood pressure regulator, is crucial to mitigate COVID-19 infections, as Guan et al. [26] validated that the single highest risk factor in infections is hypertension in 15% of the 1099 COVID-19 patients that participated in the study.

### 3.1. Vitamin D and the SARS-CoV-2 Infection

An uncontrolled inflammatory response to SARS-CoV-2 is the major cause of disease severity and death in COVID-19 patients [27]. This response is associated with high levels of circulating cytokines, tumor necrosis factors (TNF), monocyte chemoattractant protein 1 (CCL2), C-reactive protein (CRP), and ferritin. Notably, Metadichol [14] inhibits CCL2 (also known as MCP-1), TNF, NF-*κ*B, and CRP, which is a surrogate marker of cytokine storms [28], and all these cytokines are increased in patients with vitamin D deficiency.

Vitamin D3 is produced in the skin upon exposure to ultraviolet B radiation via the generation of 7-dehydrocholesterol followed by a thermal reaction. It is converted to 25(OH)D in the liver and subsequently to 1,25(OH)2D (calcitriol) in the kidneys, where calcitriol binds to the nuclear vitamin D receptor (VDR). This receptor is a DNA-binding protein that interacts with regulatory sequences near target genes and recruits chromatin active complexes that genetically and epigenetically regulate the gene transcripts [29]. Vitamin D reduces the risk of infections by mechanisms that induce cathelicidin and defensins [30], resulting in lowered replication rates of viruses and reduced concentrations of proinflammatory cytokines [31]. For instance, supplementation with 4000 IU/d of vitamin D decreased the dengue virus infection [32]. Inflammatory cytokine levels increase in viral and bacterial infections, as observed in COVID-19 patients. However, vitamin D can reduce the production of proinflammatory cytokines, such as TNF and interferon (IFN), secreted by T helper type 1 (Th1) cells [33] and thus is a modulator of adaptive immunity [34]. For example, it primarily suppresses Th1-mediated immune responses by repressing the production of the inflammatory cytokines interleukin- (IL-) 2 and IFN-gamma [35]. Additionally, 1,25(OH)2D3 promotes cytokine production by T helper type 2 (Th2) cells and enhances the indirect suppression of Th1 cells by promoting the actions of a multitude of cell types [36]. It also induces the expression of T regulatory cells, thereby inhibiting inflammatory processes [37]. Remarkably, Metadichol is an inverse agonist (protean agonist) [14] of VDR; i.e., it binds to VDR at the same site as calcitriol but has different properties. It is the only known inverse agonist of VDR in medical literature.

### 3.2. Telomerase and Viral Infections

Viral infection places substantial strain on the body. Notably, CD8+ T cells mediate adaptive immunity [38] to protect the body from microbial invaders. However, they can easily reach their Hayflick limit due to progressive telomere shortening [39]; this is more likely if the telomeres are already short. Thus, infections can enormously strain the immune cells to replicate. Naive T and B cells [40, 41] are particularly important for protection against new pathogens, such as SARS-CoV-2. Thus, the quantity of these cells is crucial to initiate an effective immune response. In this regard, 1 pico gram/mL of Metadichol has been found to increase h-TERT (telomerase) expression by 16-fold [42].

### 3.3. Aryl Hydrocarbon Receptor and Viral Infections

One of the major complications observed in infected COVID-19 patients is respiratory failure. A possible underlying mechanism is the activation of the aryl hydrocarbon receptor (AHR) during COVID-19 that can impact antiviral immunity and the function of repair-associated lung cells [43]. Thus, the AHR signaling pathway can dampen the immune response against SARS-CoV-2 [44]. Remarkably, studies have reported that while AHR signaling is required for SARS-CoV-2 replication, upregulation of this pathway may be deleterious to the virus. This is because AHR limits activation and interferes with multiple antiviral immune mechanisms, including IFN-I production and intrinsic immunity [45] which suggests that AHR signaling constrains IFN type I-mediated innate antiviral defense and the need to block constitutive AHR activity. Of note, only an inverse agonist can hinder this activity. Previously, we have shown that Metadichol® binds to AHR as an inverse/protean agonist [46] and thus reduces complications attributed to uncontrolled inflammation and cytokine storms.

### 3.4. Vitamin C and Viral Infections

There is a need to boost the innate and adaptive immunity of a person in response to infectious diseases. Micronutrients that have been identified to robustly promote immunity are vitamins C and D. Vitamin C is essential for a healthy and functional host defense, and its pharmacological application has been demonstrated to enhance immune function [47]. It exhibits antiviral properties that inhibit the replication of HSV-1, poliovirus type 1, influenza virus type A and B [48], and rabies virus *in vitro* [49].

Vitamin C deficiency reduces cellular [50–54] and humoral immune responses. Treatment of healthy subjects promotes and enhances natural killer (NK) cell activities [55], underlining the immunological importance of vitamin C [56, 57]. This validates its crucial role in various aspects of immune cell functions, such as immune cell proliferation and differentiation, in addition to its anti-inflammatory properties. Vitamin C is also required as a cofactor for the optimal activity of newly characterized hydroxylase enzymes, which regulate the activity, gene transcription, and signaling of hypoxia-inducible factors in immune cells [58–60]. Of note, studies have demonstrated that Metadichol administration increases the endogenous vitamin C levels by recycling it to levels that are not achieved by oral intake, and these levels bring about changes in improving diverse biomarkers [61–63].

### 3.5. Gene Cluster Network Analysis in COVID-19 Infections

The present drug discovery paradigm is based on the idea of one gene, one target, and one disease. Nevertheless, it has become clear that it is difficult to achieve single-target specificity. Thus, it is more likely that targeting multiple genes rather than single genes can help block multiple paths of disease progression [64, 65]. Gene network analysis provides a minimum set of target genes that form the basis of a disease. This cluster of genes modulates gene pathways and biological networks involved in the disease. The database http://www.ctdbase.org [66] was used to curate genes that were relevant to COVID-19 (Table 9).  Table 10 lists the curated genes and the diseases that they are involved in.

**Figure 7:**
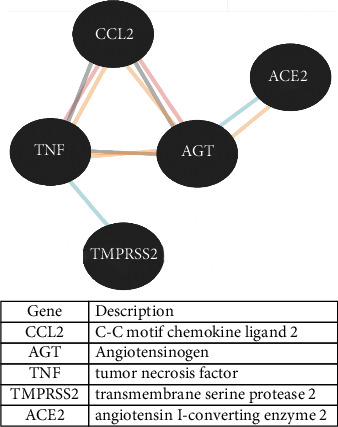
Potential key gene targets in the SARS-CoV-2 infection.

**Figure 8:**
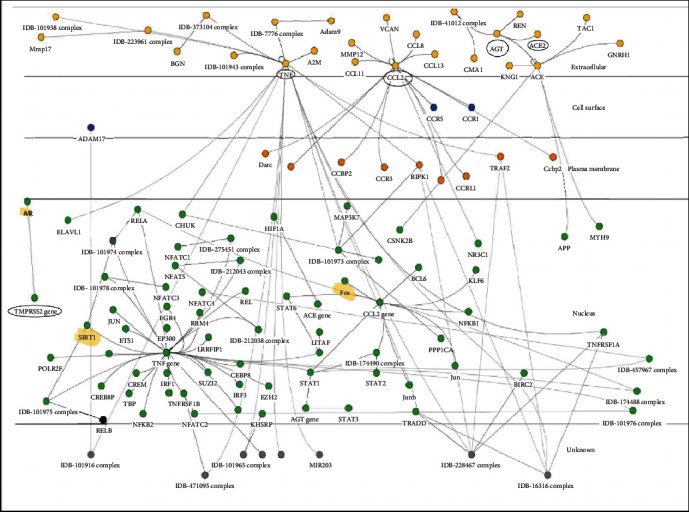
Network analysis of the genes involved in the SARS-CoV-2 infections.

The 13 identified genes were screened and categorized in set of five genes: *TNF*, *CCL2*, *ACE2*, *TMPRSS2*, and *AGT*, 0000which belong to the renin-angiotensin system network (Figure 7). Metadichol modulated all these genes by binding to VDR. A similar analysis of these genes demonstrated that they were clustered closely in diseases and had a highly significant *p* value < 10^−6^. Furthermore, a network of these five closely related genes was generated using http://www.innatedb.org [67] (Figure 8). This analysis integrates known gene interactions and pathways curated from major public databases. The highlighted ones in yellow are SIRT1, androgen receptor (AR), and FOS.

**Figure 9:**
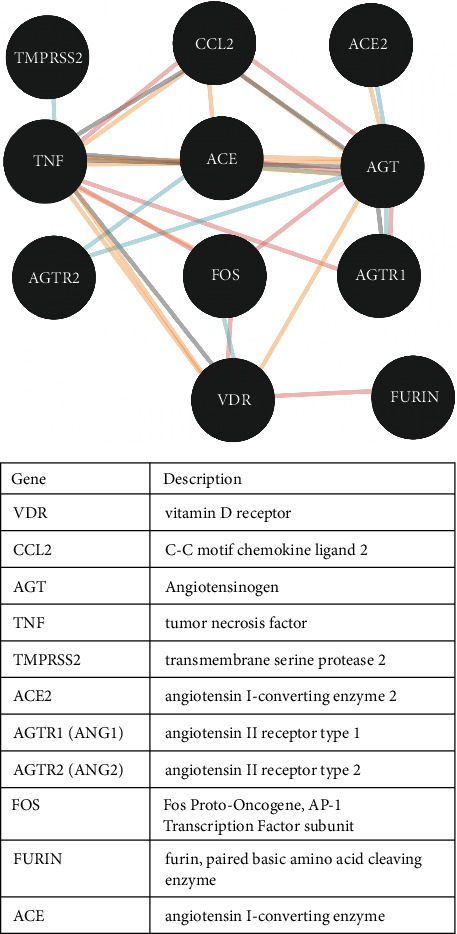
Network of relationship between the vitamin D receptor and RAAs.

Glinsky [68] suggested that vitamin D is a potential mitigation agent that prevents SARS-CoV-2 entry. Notably, Metadichol binds to VDR which controls the expression of FOS [69]. Moreover, VDR regulates SIRT1 [70] in viral infections [71]. Subsequently, SIRT1 regulates the expres00000sion of AR [72] that in turn regulates the expression of TMPRSS2.  Figure 9, generated using PACO [73], presents the gene network and corresponding regulatory relationships. The analysis revealed that VDR also regulated FOS expression, whereas FOS regulated AGT expression and AGT mediated the expression levels of AGTR1 and ACE.

Wambier and Goren [74] suggested that the SARS-CoV-2 infection is likely to be androgen-mediated as AR controls the expression of TMPRSS2. The first step that occurs in the COVID-19 infection is the priming of the SARS-CoV-2 spike proteins by TMPRSS2; these proteins cleave ACE2 to augment viral entry into the host cells. However, Metadichol can completely inhibit this key priming step.

Proteases such as furin [75] and Adam-17 have been reported to activate the spike protein *in vitro*, enabling viral spread and pathogenesis in infected hosts. Notably, VDR controls furin expression via its interaction with SRC (proto-oncogene tyrosine-protein kinase Src) [76]. On the other hand, Adam-17 is regulated via CEPBP (CCAAT Enhancer Binding Protein Beta) [77, 78], which is involved in the regulation of genes involved in immune and inflammatory responses. Recently, Ulrich and Pillat [79] proposed that CD147, like ACE2, is another host receptor used by the virus to enter host cells. CD147 is a known receptor [80] of *Plasmodium falciparum*, the parasite that causes malaria in humans. Remarkably, a previous study has demonstrated that Metadichol [14] (US patent 9,006,292) inhibits malarial parasites.

### 3.6. Controlling Cytokine Storms

**Figure 10:**
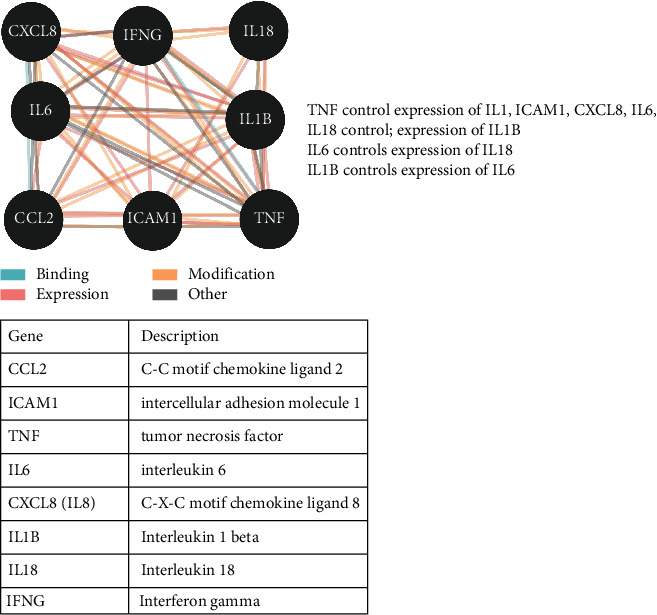
Gene-cytokine relationships.

**Figure 11:**
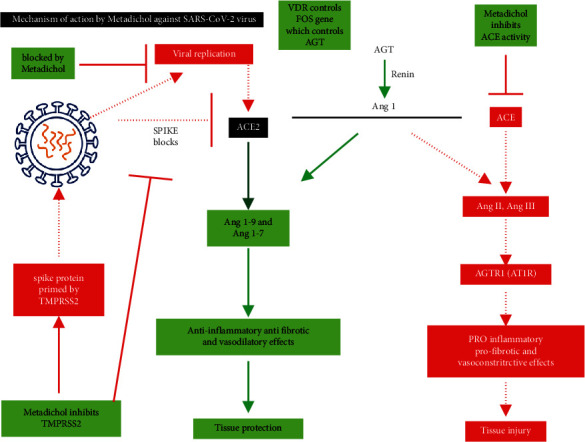
Mechanism of action of Metadichol against the SARS-CoV-2 virus.

A cytokine storm develops when an initial immune response induces the production of cytokines. It is initiated in the host body in response to SARS-CoV-2 and leads to inflammation and increases the secretion of the proinflammatory cytokines.  Figure 10 depicts the cytokine relationship network generated in this study using PACO. Cytokines can activate T cells and cause tissue damage and infection in the lungs. Remarkably, Metadichol is an *in vivo* inhibitor [14] (US patent 8,722,093) of TNF alpha. The endocytosis of ACE2 with SARS-CoV-2 results in a reduction in ACE2 on cell surfaces, thus increasing serum angiotensin II levels [81]. Angiotensin II is a vasoconstrictor and proinflammatory cytokine (Figure 11) that acts via AT1R [82]. The angiotensin II-AT1R axis leads to a proinflammatory state [83] in the host, causing infections by activating NF-*κ*B and increasing IL-6 levels in multiple inflammatory and autoimmune diseases [84].

Thus, the dysregulation of angiotensin II downstream of ACE2 leads to cytokine release in COVID-19 patients. This increases TNF levels that in turn elevate IL-6, CCl2, and CRP levels. Therefore, cytokine storms [85] result in ARDS. However, Metadichol is an ACE inhibitor that blocks the angiotensin I and II pathways, promoting an anti-inflammatory state.

### 3.7. Clinical Setting

A pilot study conducted by a third party, Kasturba Hospital in Mumbai, India, on 30 COVID-19 patients with minor symptoms revealed that Metadichol treatment (20 mg/day) eliminated by the Rt-PCR test the 000000virus in 75% of patients after four days of treatment (supplements available (here)). To validate this finding, a larger study consisting of a Metadichol treatment group and control groups with only standard care provided to the participants was initiated. We hope to communicate these results in the future.

## 4. Summary and Conclusions

Metadichol inhibits the entry of SARS-CoV-2 into host cells by inhibiting TMPRSS2, thus allowing ACE2 to play a critical role in the renin-angiotensin pathway. In addition, it enhances the antiviral response of the host by increasing the innate and adaptive immune responses through the vitamin D pathway and by endogenously increasing the vitamin C levels. In addition, telomerase activity also plays a key role in maintaining the levels of naive T and B cells required to fight infections. Metadichol modulates cytokine storms, as it is an inhibitor of TNF, ICAM1, and CCL2 that play a key role in generating cytokine storms. Metadichol also regulates COVID-19-associated comorbidities [86, 87], such as hypertension and diabetes [88–90]. Thus, Metadichol has the potential to improve the long-term prognosis of the affected patient population. Metadichol acts on multiple genes and has over 2000 unique gene interactions, thereby resulting in a network that brings about homeostasis and prevents SARS-CoV-2 infections.

Metadichol is a safe, noncytotoxic product. LD_50_ is greater than 5000 mg per kilo in rat studies. It is made from renewable sources like sugar cane or rice. It has been commercially available for the last six years and has no reported side effects. Thus, Metadichol can potentially be used as an immune modulator to prevent future occurrences of SARS-CoV-2 and possibly other predicted infections, facilitating a rapid return to normal social and economic human activities worldwide.

## Figures and Tables

**Table 1:** *In vitro* antiviral assay.

	CC_50_	EC_90_	SI_90_
Metadichol (*μ*g/mL)	4	0.15	20
M128533 (*μ*g/mL)	>10	0.2	>33

CC_50_: 50% cytotoxic concentration of compound without virus added; EC_50_: 50% effective antiviral concentration; EC_90_: calculated concentration to reduce viral yield by 1 log (90%); SI: CC_50_/EC_50_.

**Table 2:** Cytotoxicity and viral yield data for each concentration of Metadichol tested.

Metadichol concentration (*μ*g/mL)	Cytotoxicity (%)	Viral titer (CCID_50_ per 0.1 mL)
100	100%	<0.7
32	100%	<0.7
10	83%	<0.7
3.2	54%	0.7
1	17%	4.3
0.3	26%	1.5
0.1	26%	5.7
0.03	26%	5.3

CCID: cell culture infectious dose (50%/mL).

**Table 3:** Raw data of Metadichol cytotoxicity in viral absence, as measured by using the neutral red assay. Units are *μ*g/mL unless noted.

Metadichol (*μ*g/mL)	Adenovirus	Tacaribe	Rift valley	SARS	Japanese encephalitis	West Nile virus	Yellow fever Powassan virus	
500	95%	98%	96%	96%	100%	100%	100%	100%
160	92%	98%	96%	95%	100%	100%	100%	100%
50	90%	97%	97%	95%	100%	100%	100%	100%
16	85%	95%	81%	92%	88%	77%	98%	100%
5	0%	23%	26%	35%	33%	28%	35%	44%
1.6	0%	2%	10%	15%	12%	14%	19%	6%
0.5	0%	3%	9%	0%	2%	3%	2%	0%
0.16	0%	17%	3%	0%	0%	0%	4%	0%
CC_50_	9.90	7.30	8.40	6.70	7.20	8.50	5.00	5.1

CC_50_: 50% cytotoxic concentration of compound without virus added.

**Table 4:** Antiviral assay of Metadichol against various viruses, as measured using the neutral red assay.

Metadichol (*μ*g/mL)	Adenovirus	Tacaribe	Rift valley fever	SARS	Japanese encephalitis	West Nile	Yellow fever	Powassan
5	100%	31%	100%	0%	56%	84%	70%	53%
1.6	100%	69%	100%	52%	87%	100%	73%	100%
0.5	100%	97%	100%	100%	100%	100%	95%	100%
0.16	100%	100%	100%	100%	100%	100%	96%	100%
EC_50_	>9.9	2.8	>8.4	1.7	>7.2	>8.5	>5	>5.1

EC_50_: 50% effective antiviral concentration.

**Table 5:** List of viruses inhibited by Metadichol *in vitro.*

Adenovirus	Rift valley
Japanese encephalitis	Marburg
Tacaribe	SARS (severe acute respiratory syndrome)
Powassan	Respiratory syncytial virus
Zika	Chikungunya
Ebola	Influenza A (H1N1)
Yellow fever	Dengue
West Nile virus	HIV (human immunodeficiency virus)

**Table 6:** TMPRSS2 assay data.

Sample	Concentration	RFU	% inhibition	IC_50_
Control	0	43233358	0.00	

Metadichol (ng/mL)	1.56	41305150	4.46	96.65 ng/mL
	3.12	39329385	9.03	
	6.25	36713767	15.08	
	12.5	33778222	21.87	
	25	30695684	29.00	
	50	26087008	39.66	
	100	16009312	62.97	

Camostat mesylate (*μ*g/mL)	0.78	37984828	12.14	26.46 *μ*g/mL
	1.56	35235186	18.50	
	3.125	31685728	26.71	
	6.25	29234396	32.38	
	12.5	23276839	46.16	
	25	18931887	56.21	
	50	8797988	79.65	

**Table 7:** ACE2 assay data.

Sample	Concentration (*μ*g/mL)	RFU	% inhibition	IC_50_ (*μ*g/mL)
Control	0	308315546	0.00	

Metadichol	0.125	290309918	5.84	30.15
	0.25	260064163	15.65	
	0.5	249149792	19.19	
	1	240301136	22.06	
	10	212275253	31.15	
	20	187702504	39.12	
	40	139821100	54.65	

DX600	0.0156	252855648	17.99	0.1027
	0.031	231028864	25.07	
	0.0625	193810784	37.14	
	0.125	145881248	52.68	
	0.25	127485752	58.65	
	0.5	111498760	63.84	

**Table 8:** ACE assay data.

	Concentration (ng/mL)	Percent inhibition
Captopril (control)	0.63	6.12
	1.25	15.04
	2.50	32.42
	5.00	43.5
	10.00	57.2
	20	76.51

Metadichol	3.9	4.61
	7.8	6.37
	15.6	14.28
	31.25	22.59
	62.5	36.71
	125	54.89
	250	60.51
	500	66.23
	1000	78.1

**Table 9:** Disease network of the 13 curated genes.

Disease name	Disease categories	Corrected *p* value	Annotated gene quantity	Annotated genes
COVID-19	Respiratory tract disease, viral disease	3.10*E* − 47	13	ACE2, AGT, CCL2, CCL3, CSF3, CXCL10, IL10, IL2, IL2RA, IL6, IL7, TMPRSS2, TNF
Pneumonia, viral	Respiratory tract disease, viral disease	4.34*E* − 46	13	ACE2, AGT, CCL2, CCL3, CSF3, CXCL10, IL10, IL2, IL2RA, IL6, IL7, TMPRSS2, TNF
Coronaviridae infections	Viral disease	1.74*E* − 44	13	ACE2, AGT, CCL2, CCL3, CSF3, CXCL10, IL10, IL2, IL2RA, IL6, IL7, TMPRSS2, TNF
Coronavirus infections	Viral disease	1.74*E* − 44	13	ACE2, AGT, CCL2, CCL3, CSF3, CXCL10, IL10, IL2, IL2RA, IL6, IL7, TMPRSS2, TNF
Nidovirales infections	Viral disease	1.74*E* − 44	13	ACE2, AGT, CCL2, CCL3, CSF3, CXCL10, IL10, IL2, IL2RA, IL6, IL7, TMPRSS2, TNF
RNA virus infections	Viral disease	4.92*E* − 27	13	ACE2, AGT, CCL2, CCL3, CSF3, CXCL10, IL10, IL2, IL2RA, IL6, IL7, TMPRSS2, TNF
Virus diseases	Viral disease	1.73*E* − 25	13	ACE2, AGT, CCL2, CCL3, CSF3, CXCL10, IL10, IL2, IL2RA, IL6, IL7, TMPRSS2, TNF
Sexually transmitted diseases, viral	Viral disease	1.38*E* − 12	7	CCL2, CCL3, IL10, IL2, IL2RA, IL6, TNF
HIV infections	Immune system disease, viral disease	1.56*E* − 12	7	CCL2, CCL3, IL10, IL2, IL2RA, IL6, TNF
Lentivirus infections	Viral disease	1.56*E* − 12	7	CCL2, CCL3, IL10, IL2, IL2RA, IL6, TNF
Retroviridae infections	Viral disease	1.56*E* − 12	7	CCL2, CCL3, IL10, IL2, IL2RA, IL6, TNF
HIV wasting syndrome	Immune system disease, metabolic disease, nutrition disorder, viral disease	4.00*E* − 04	2	IL6, TNF
Coxsackievirus infections	Viral disease	0.001	2	IL6, TNF
Enterovirus infections	Viral disease	0.0044	2	IL6, TNF
Picornaviridae infections	Viral disease	0.00519	2	IL6, TNF

**Table 10:** Disease network of genes implicated in the SARS-CoV-2 infection.

Disease name	*p* value	Corrected *p* value	Genes	Annotated genes
COVID-19	1*E* − 18	5.44*E* − 16	5	ACE2, AGT, CCL2, TMPRSS2, TNF
Pneumonia, viral	1.56*E* − 18	8.46*E* − 16	5	ACE2, AGT, CCL2, TMPRSS2, TNF
Coronaviridae infections	3.4*E* − 18	1.85*E* − 15	5	ACE2, AGT, CCL2, TMPRSS2, TNF
Coronavirus infections	3.4*E* − 18	1.85*E* − 15	5	ACE2, AGT, CCL2, TMPRSS2, TNF
Nidovirales infections	3.4*E* − 18	1.85*E* − 15	5	ACE2, AGT, CCL2, TMPRSS2, TNF
Pneumonia	9.42*E* − 15	5.11*E* − 12	5	ACE2, AGT, CCL2, TMPRSS2, TNF
Respiratory tract infections	3.13*E* − 13	1.7*E* − 10	5	ACE2, AGT, CCL2, TMPRSS2, TNF
RNA virus infections	2.46*E* − 12	1.34*E* − 09	5	ACE2, AGT, CCL2, TMPRSS2, TNF
Virus diseases	9.48*E* − 12	5.15*E* − 09	5	ACE2, AGT, CCL2, TMPRSS2, TNF

## Data Availability

All data are cited in the document.

## Supplementary Materials

Supplementary Materials Describes the neutralization assays of Metadichol performed with HEK 293T-ACE2, a human embryonic kidney cell line 00overexpressing ACE2, the receptor of the SARS-CoV-2 0virus.
