# Supplementary material for: Metadichol®: A Novel Nanolipid Formulation That Inhibits SARS-CoV-2 and a Multitude of Pathological Viruses In Vitro
Source: Biomed Res Int. 2022 Jan 15;2022:1558860. doi: 10.1155/2022/1558860 (PMC8760534; doi:10.1155/2022/1558860)
Supplement: Supplementary Materials — Describes the neutralization assays of Metadichol performed with HEK 293T-ACE2, a human embryonic kidney cell line 00overexpressing ACE2, the receptor of the SARS-CoV-2 0virus. [file 1558860.f1.zip › Description-supplementary material.pdf]

Summary of Supplementary Material

The SARS-CoV-2 Spike Pseudo-typed Lentivirus were produced with SARS-CoV-2 Spike (Genbank Accession #QHD43416.1) as the envelope glycoproteins instead of the commonly used VSV-G. These pseudo-virions also contain the firefly luciferase gene driven by a CMV promoter therefore, the spike-mediated cell entry can be conveniently measured via luciferase reporter activity

Neutralization assays were performed using a non-replicative VSV pseudo-virus with a firefly luciferase reporter gene and also carrying the spike protein of SARS-CoV-2 (SARS-CoV-2/VSV-Luc pseudo-virus). Metadichol was evaluated with a similar VSV pseudo-virus reporter carrying the envelope glycoprotein (VSVg) of the vesicular stomatitis virus (VSVg/VSV-Luc pseudovirus).

The neutralization assays were performed with HEK 293T-ACE2, a human embryonic kidney cell line overexpressing ACE2, the receptor of SARS-CoV-2 virus
