# Supplementary material for: Metadichol®: A Novel Nanolipid Formulation That Inhibits SARS-CoV-2 and a Multitude of Pathological Viruses In Vitro
Source: Biomed Res Int. 2022 Jan 15;2022:1558860. doi: 10.1155/2022/1558860 (PMC8760534; doi:10.1155/2022/1558860)
Supplement: Supplementary Materials — Describes the neutralization assays of Metadichol performed with HEK 293T-ACE2, a human embryonic kidney cell line 00overexpressing ACE2, the receptor of the SARS-CoV-2 0virus. [file 1558860.f1.zip › Supplementay-material.pdf]

Supplementary Material

Results of neutralization assays of Metadichol performed with HEK 293T-ACE2, a human embryonic kidney cell line over expressing ACE2, the receptor of SARS-CoV-2 virus.

Neutralization Activity Metadichol ® Against SARS-CoV-2 Pseudovirus

Background to Neutralization assay

Source: https://bpsbioscience.com/ace

Commercial contract work outsourced by a service provider;

Retrovirox,Inc San Diego USA

In Viral replication, the virus attaches to the host cell surface before entering the cell. The viral Spike protein recognizes and attaches to the Angiotensin-Converting Enzyme 2 (ACE2) receptor found on the surface of type I and II pneumocytes, endothelial cells, and ciliated bronchial epithelial cells. Drugs targeting the interaction between the Spike protein of SARS-CoV-2 and ACE2 may offer protection against the viral infection.

SARS-CoV2 Pseudovirus					VSVg Pseudovirus		
Sample	IC50 (µg/mL)	Assay S/B*	C.V.[#]		IC50 (µg/mL)	Assay S/B*	C.V.[#]
metadichol	6.7	538	1.7%		3.4	297	1.0

The SARS-CoV-2 Spike Pseudo-typed Lentivirus were produced with SARS-CoV-2 Spike (Genbank Accession #QHD43416.1) as the envelope glycoproteins instead of the commonly used VSV-G. These pseudovirions also contain the firefly luciferase gene driven by a CMV promoter therefore, the spike-mediated cell entry can be conveniently measured via luciferase reporter activity

Neutralization assays were performed using a non-replicative VSV pseudovirus with a firefly luciferase reporter gene and also carrying the spike protein of SARS-CoV-2 (SARS-CoV-2/VSV-Luc pseudovirus). Metadichol was evaluated with a similar VSV pseudovirus reporter carrying the envelope glycoprotein (VSVg) of the vesicular stomatitis virus (VSVg/VSV-Luc pseudovirus).

The neutralization assays were performed with HEK 293T-ACE2, a human embryonic kidney cell line overexpressing ACE2, the receptor of SARS-CoV-2 virus. Test-item was pre-incubated for 60 min at 37°C with a previously titrated inoculum of pseudovirus. Then, the mixture was added to 293T-ACE2 cells. Twelve hours later one volume of complete tissue culture media (DMEM supplemented with 10% FBS) was added to the cells. Neutralization of pseudovirus entry was determined by measuring firefly luciferase activity (RLUs) 12h after addition of media (24h post-infection). Putative neutralizing agents were present in the cell culture for the duration of the experiment. Eight concentrations of theMetadichol samples were tested in duplicates utilizing 10-fold serial dilutions started at 10µg/mL.

Neutralization Activity of Test-Item metadichol

At the highest concentration tested (10µg/mL) metadichol inhibited infection with VSV pseudoviruses carrying SARS-CoV-2 spike. The IC50 neutralization values for the test-item is shown in Table 1 below.

Inhibitors and Quality Controls

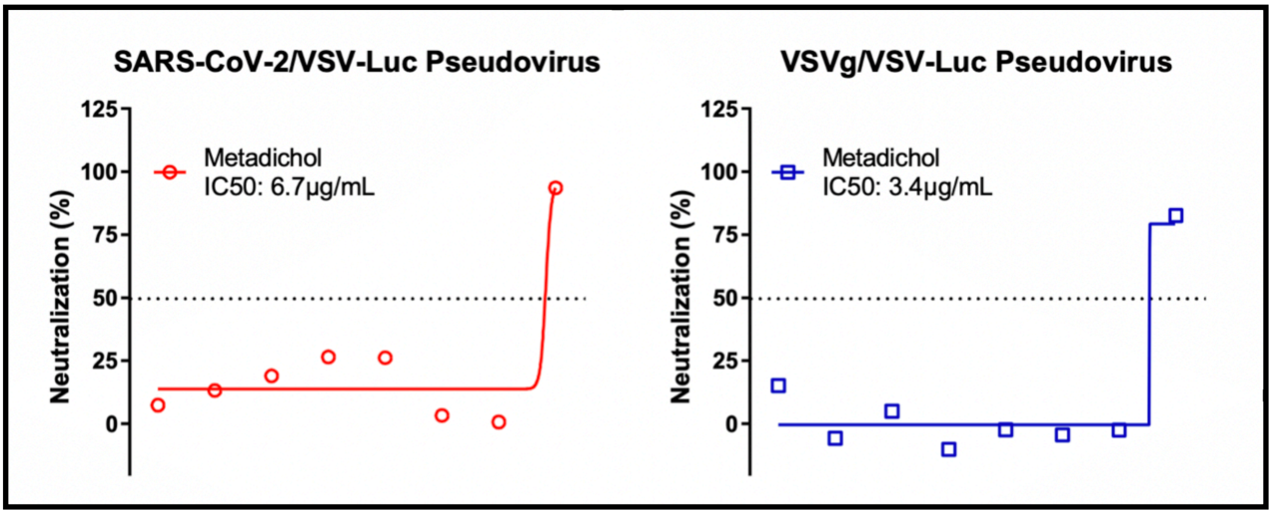

Determination of IC50 values for pseudovirus entry. Values indicate the percentage of SARS-CoV-2/VSV-Luc or VSVg/VSV-Luc pseudovirus neutralization as compared to samples incubated with no test-item (DMEM 2% FBS, "vehicle alone"). Results show the average of duplicate data points. Data was adjusted to a sigmoid function and IC50 values were calculated using

Quality controls for the pseudovirus assays were performed on every plate to determine: i) signal to background (S/B) values; ii) inhibition by a known inhibitor of SARS-CoV-2 entry, and iii) variation of the assay, as measured by the coefficient of variation (C.V.) of all data points.

All controls worked as anticipated for each assay. A recombinant form of the spike glycoprotein receptor binding domain (RBD) from SARS-CoV-2 (Wuhan-Hu-1) neutralized SARS-CoV-2 VSV pseudovirus (76 percent inhibition when tested at 25µg/mL). A rabbit polyclonal antiserum against a truncated form of SARS-CoV spike protein also neutralized SARS-CoV-2 VSV pseudovirus (67 percent inhibition when tested at 1:80 dilution). By contrast, the recombinant spike protein diminished infection of the pseudovirus carrying VSVg by only 20 percent at the same concentration, whereas the 1:80 dilution of polyclonal anti-SARS spike displayed no inhibition on the VSVg pseudovirus.

Infection with SARS-CoV-2 or VSVg carrying pseudoviruses resulted in a luciferase signal 538-fold, or 297-fold greater than the signal generated by a similar pseudovirus lacking envelope glycoproteins ("No-Env"). When the signal of SARS-CoV-2 or VSVg carrying pseudoviruses was compared to uninfected cells, the increase was 236-fold and 358-fold, respectively. These findings demonstrate that the signal generated by the pseudovirus is the result of entry mediated by either the Spike or VSVg proteins.

Summary of Results.
IC50 (neutralization) values are shown for each test-item and assay. Signal-to-background ratios (S/B), and average coefficients of variation (C.V.) of duplicate data-points for which 50% or greater infection (RLUs) was observed as compared to cells infected with pseudovirus in the absence of test-item (neutralization assay). When neutralization of pseudovirus infection was below 50% IC50 values are shown as greater than highest concentration tested.

*Signal to background level was calculated by dividing the signal in uninfected cells ("mock-infected"), by the signal in cells challenged with

SARS-CoV-2/VSV-Luc or VSVg/VSV pseudovirus. #C.V. for the assays was calculated as the average of C.V. values determined for all data points displaying neutralization of 50% or greater as compared to cells infected in the presence of vehicle alone.
